# En Face OCT Imaging for the Diagnosis of Outer Retinal Tubulations in Age-Related Macular Degeneration

**DOI:** 10.1155/2012/542417

**Published:** 2012-08-30

**Authors:** Benjamin Wolff, Alexandre Matet, Vivien Vasseur, José-Alain Sahel, Martine Mauget-Faÿsse

**Affiliations:** Rothschild Ophthalmologic Foundation, 25 rue Manin, 75019 Paris, France

## Abstract

*Purpose*. “En face” is an emerging imaging technique derived from spectral domain optical coherence tomography (OCT). It produces frontal sections of retinal layers, also called “C-scan OCT.” Outer retinal tubulations (ORTs) in age-related macular degeneration (AMD) are a recent finding evidenced by spectral-domain OCT. The aim of this study is to characterize the morphology of ORT according to the form of AMD, using “en-face” spectral domain OCT. *Methods*. “En face” OCT imaging was prospectively performed in 26 consecutive eyes with AMD that also had ORT. *Results*. There were 15 neovascular, 8 atrophic, and 3 eyes with a mixed (fibrotic and atrophic) form of AMD. Among the neovascular group, the most frequent tubulation pattern on “en-face” OCT was a branching network emanating from a fibrovascular scar; we term this pattern as “pseudodendritic.” It did not require treatment when observed as an isolated finding. In all cases of atrophic AMD, the tubular network was located at the edge of the geographic atrophy area, and formed a “perilesional” pattern. Six atrophic cases showed tubular invaginations inside this area. *Conclusion*. “En face” OCT is a valuable technique in the diagnosis and followup of macular disease. It revealed the main characteristic patterns of ORT associated with neovascular and atrophic AMD.

## 1. Introduction

Outer retinal tubulations (ORT) have been recently identified in age-related macular degeneration (AMD) thanks to technological improvements in Spectral-Domain Optical Coherence Tomography (SD-OCT) [1]. ORT usually have a characteristic presentation, and thus can be easily diagnosed. It is of clinical significance to recognize them, since they do not indicate ongoing exudative process and, therefore, do not require treatment.

On B-scans ORT are round hyporeflective lesions, which may contain a few focal hyperreflective spots, and are always delineated by an hyperreflective ring, in contrast to the completely hyporeflective retinal cystoid lesions. They are always located at the level of the outer nuclear layer, and in AMD are classically found very close to areas of neovascular fibrosis or retinal atrophy. These lesions have been named “tubulations” because they exhibit a tubular morphology when observed in frontal sections using “en face” OCT scans, also called C-scans.

This study aims to differentiate between the ORT presentations observed by “en face” OCT in exudative and atrophic AMD.

## 2. Methods

Eyes with a diagnosis of neovascular or atrophic AMD, and demonstrating ORT on SD-OCT, were prospectively studied using “en face” OCT. For all cases, systematic work-up included macular examination by SD-OCT (Spectralis Heidelberg Engineering, Heidelberg, Germany) and macular mapping consisting of 197 transverse sections in a 5.79 × 5.79 mm^2^ central retinal area. Tridimensional reconstruction generated by the pooling of these sections provided a virtual macular brick, through which 496 shifting sections in the coronal plane resulted in C-scan, or “en face” OCT, while B-scan, or conventional OCT, is derived from sagittal and transverse sections. These results were then compared with data from classical retinal imaging, namely, fundus photography and angiography. For all eyes with atrophic AMD, autofluorescence imaging was also performed.

## 3. Results

Twenty-six eyes of 23 AMD patients demonstrating ORT on B-scan SD-OCT were analyzed by “en face” OCT. Neovascular AMD with a neovascular fibrotic scar was identified in 15 eyes, geographic atrophy in 8 eyes and “mixed” exudative and atrophic AMD form in 3 eyes. 

**Figure 1:**
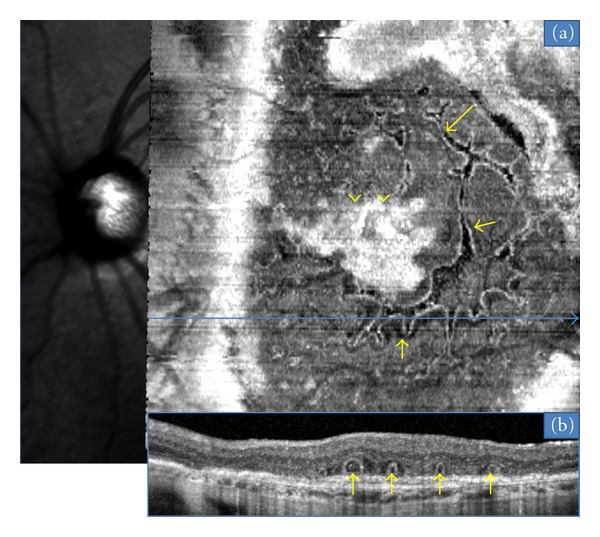
ORT (arrows) located above a fibrovascular scar (arrows heads). A branching network is observed emanating from hyper reflective the fibrovascular scar with numerous ramifications (or “pseudodendritic” pattern) is observed with “en face” OCT (a). Corresponding B-scan (b) demonstrates multiples ORT.

**Figure 2:**
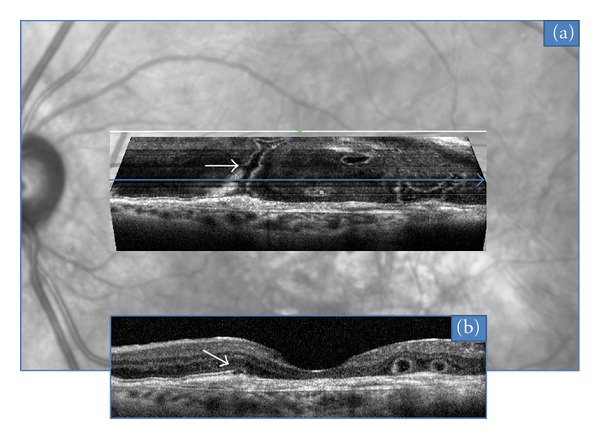
Tubular (arrows) pattern of ORT located above a fibro-vascular scar, observed with “en face” OCT (a). Corresponding B-scan OCT (b).

**Figure 3:**
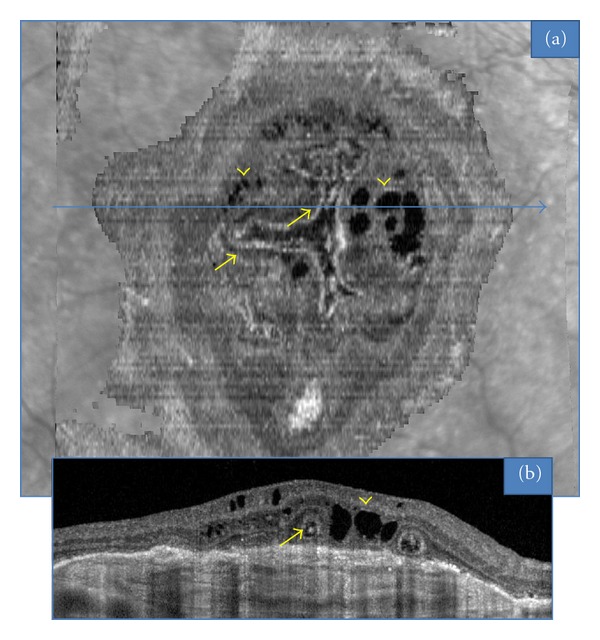
ORT (arrows) associated with intraretinal cystoid cavities (arrow heads) in the case of a fibro-vascular scar, observed with “en face” OCT (a). Corresponding B-scan OCT (b).

In the 15 neovascular fibrotic scar cases, the most frequent finding regarding ORT was a branching network emanating from a fibrovascular scar (*n* = 11) with numerous ramifications (or digitations) resulting in a “pseudodendritic” pattern (Figure 1). In all 4 remaining eyes, ORT had a tubular shape, appearing round in B-scans, with no or limited digitations (Figure 2). The fibrotic neovascular choroidal network was formally identified in all 15 eyes as a hyperreflective lesion above the level of the retinal pigment epithelium. Associated intraretinal cystoid cavities related to neovascular reactivation or the progression of retinal degeneration were observed in 7 cases (Figure 3).

**Figure 4:**
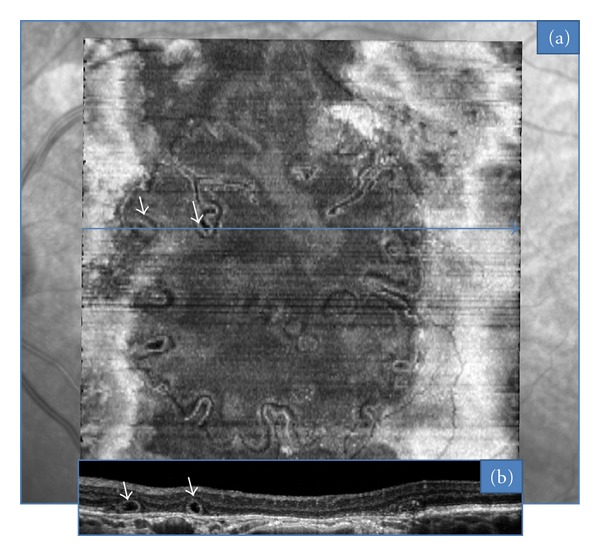
ORT observed with “en face” OCT in a case of geographic atrophy (a). ORTs follow a circular pattern associated with invaginations (arrows) inside the atrophic zone. Corresponding B-scan OCT (b).

In all the 8 geographic atrophy AMD cases, ORT also exhibited a round section on B-scan. However, C-scans showed that these tubulations followed the margins of the chorioretinal atrophic area (Figure 4). This ORT aspect was then termed “perilesional.” Correlation with autofluorescence imaging demonstrated that ORT did not extend beyond the hyperreflective border of the atrophic area. Invaginations of ORT inside the atrophic zone were also found in 6 cases.

Of the 3 eyes with “mixed” AMD, with a fibrotic and atrophic scar, one eye had cystoid lesions and invagination of the ORT network inside the atrophic area.

## 4. Discussion

The present study describes en-face OCT features of outer retinal tubulations in wet and dry AMD. In this small series, most of the exudative AMD patients with ORT exhibit a similar branching pattern resembling a dendritic cell that we called therefore “pseudodendritic”. In atrophic AMD cases, ORTs also exhibit a common pattern around the margin of the atrophic zone, that we called “perilesional.”

“En face” OCT imaging is a technique that has been used for almost ten years [2] although it still has limited application in AMD. Nonetheless, this technique enables to assess the extent of structural damage occurring in AMD. The observation of ORTs, using “en face” OCT, also indicates the size of these lesions inside the retina. ORT can be frequently found among AMD patients (56% of exudative and 21% of atrophic forms) [3], and their incidence is probably underestimated [4]. A combination of SD-OCT with “en face” OCT enhances its sensitivity, allowing earlier diagnosis and a more reliable followup. This technique also improves the distinction between ORT and their main differential diagnosis: cystoid cavities and forms of serous retinal detachments. 

Physiopathogenic mechanisms leading to ORT remain poorly understood. According to Zweifel et al. [1], these lesions may result from the outward folding of the photoreceptor layer. Tissue damage associated with retinal degeneration may produce a loss of interdigitations of the photoreceptors with the retinal pigmentary epithelium, and a disruption of tight junctions between the outer segments and adjacent glial elements. Upon repeated microscopic injury, the photoreceptor layer may fold into tubular structures limited to the outer retina.

This type of degeneration is not limited to AMD. ORT have been observed in other degenerative retinal diseases, including Bietti's crystalline dystrophy [5] and Best's disease. Moreover, they have been histologically described under the term “rosette formations” in retinitis pigmentosa [6]. We had previously ventured the hypothesis of an inflammatory origin explaining ORT formation [3]. It now seems unlikely considering the extent of these lesions when viewed by “en face” OCT. The configuration of ORT observed in neovascular (pseudo-dendritic) and atrophic (perilesional) AMD strongly supports the mechanism of a tissue degeneration process causing remodeling of the outer retinal layers.

## 5. Conclusion

In conclusion, “en face” OCT is a novel technology offering a deeper insight into the understanding and followup of macular disease. This valuable technique allowed the characterization of the main subtypes of tubular formations in AMD: “pseudo-dendritic” forms that develop next to fibrotic scars and “perilesional” forms that develop in the periphery of atrophic areas. This prospective study was based on a limited number of cases. Further studies including more patients are necessary to confirm these results.
